# 2-Isonicotinoyl-*N*-phenyl­hydrazine­carbothio­amide dimethyl­formamide hemisolvate

**DOI:** 10.1107/S1600536811011950

**Published:** 2011-04-13

**Authors:** Pei-Hua Zhao, Shao-Liang Jiang

**Affiliations:** a School of Materials Science and Engineering, North University of China, Taiyuan 030051, People’s Republic of China; b College of Pharmaceutical Science, Zhejiang University of Technology, Hangzhou 310014, People’s Republic of China

## Abstract

The title compound, C_13_H_12_N_4_OS·0.5C_3_H_7_NO, contains four hydrazine mol­ecules and two solvent mol­ecules in the asymmetric unit. The dihedral angles between the pyridine and phenyl rings in the hydrazine mol­ecules are 67.51 (16), 68.28 (16), 81.36 (15) and 83.32 (15)°. In the crystal, the mol­ecules are linked by N—H⋯N, N—H⋯O and N—H⋯S hydrogen bonds.

## Related literature

For the biological activity of related compounds and further references, see: Liu *et al.* (2011 ▶).
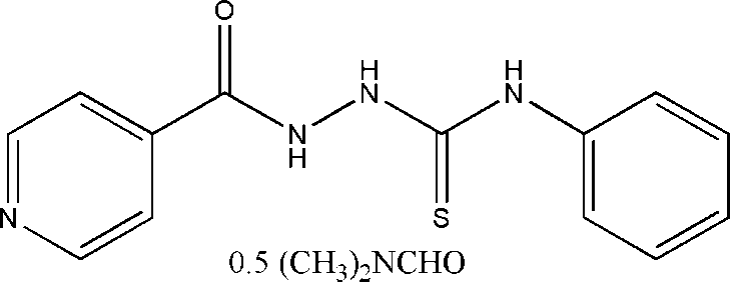


## Experimental

### 

#### Crystal data

2C_13_H_12_N_4_OS·C_3_H_7_NO
*M* *_r_* = 617.74
Triclinic, 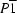

*a* = 9.901 (3) Å
*b* = 16.583 (5) Å
*c* = 18.610 (5) Å
α = 99.350 (6)°
β = 91.831 (6)°
γ = 96.454 (4)°
*V* = 2991.8 (15) Å^3^
*Z* = 4
Mo *K*α radiation
μ = 0.23 mm^−1^
*T* = 113 K
0.22 × 0.20 × 0.10 mm

#### Data collection

Rigaku Saturn CCD diffractometer
Absorption correction: multi-scan (*CrystalClear*; Rigaku/MSC, 2005 ▶) *T*_min_ = 0.952, *T*_max_ = 0.978
28071 measured reflections
10511 independent reflections
8262 reflections with *I* > 2σ(*I*)
*R*_int_ = 0.053

#### Refinement

*R*[*F* ^2^ > 2σ(*F* ^2^)] = 0.069
*wR*(*F* ^2^) = 0.143
*S* = 1.13
10511 reflections
827 parameters
12 restraints
H atoms treated by a mixture of independent and constrained refinement
Δρ_max_ = 0.80 e Å^−3^
Δρ_min_ = −0.62 e Å^−3^

### 

Data collection: *CrystalClear* (Rigaku/MSC, 2005 ▶); cell refinement: *CrystalClear*; data reduction: *CrystalClear*; program(s) used to solve structure: *SHELXS97* (Sheldrick, 2008 ▶); program(s) used to refine structure: *SHELXL97* (Sheldrick, 2008 ▶); molecular graphics: *SHELXTL* (Sheldrick, 2008 ▶); software used to prepare material for publication: *CrystalStructure* (Rigaku/MSC, 2005 ▶).

## Supplementary Material

Crystal structure: contains datablocks global, I. DOI: 10.1107/S1600536811011950/hb5821sup1.cif

Structure factors: contains datablocks I. DOI: 10.1107/S1600536811011950/hb5821Isup2.hkl

Additional supplementary materials:  crystallographic information; 3D view; checkCIF report

## Figures and Tables

**Table:** 

S1—C7	1.699 (3)
N1—C7	1.325 (4)
N1—C6	1.437 (4)
N2—C7	1.349 (4)
N2—N3	1.391 (4)

**Table:** 

C7—N1—C6	122.5 (3)
C7—N2—N3	120.9 (3)
C12—N4—C11	116.4 (3)
N1—C7—N2	117.8 (3)
N1—C7—S1	123.4 (2)
N2—C7—S1	118.7 (2)

**Table 2:** Hydrogen-bond geometry (Å, °)

*D*—H⋯*A*	*D*—H	H⋯*A*	*D*⋯*A*	*D*—H⋯*A*
N3—H3*A*⋯O6^i^	0.90 (1)	2.63 (3)	3.325 (5)	135 (3)
N2—H2*A*⋯S1^i^	0.90 (1)	2.34 (1)	3.229 (3)	171 (3)
N3—H3*A*⋯O2^ii^	0.90 (1)	2.08 (3)	2.812 (4)	138 (3)
N14—H14*A*⋯S2^ii^	0.90 (1)	2.46 (1)	3.341 (3)	166 (3)
N5—H5*A*⋯N8^iii^	0.90 (1)	2.21 (1)	3.087 (4)	165 (3)
N6—H6*A*⋯S4^iv^	0.90 (1)	2.46 (1)	3.341 (3)	167 (3)
N11—H11*A*⋯O4^iv^	0.90 (1)	2.01 (2)	2.818 (3)	149 (3)
N10—H10*A*⋯S3^v^	0.90 (1)	2.32 (1)	3.211 (3)	170 (3)
N13—H13*A*⋯N16^vi^	0.90 (1)	2.17 (1)	3.041 (4)	163 (3)
N1—H1*A*⋯N12	0.90 (1)	2.04 (1)	2.909 (4)	164 (3)
N7—H7*A*⋯O1	0.90 (1)	1.95 (2)	2.784 (4)	155 (3)
N9—H9*A*⋯N4	0.90 (1)	2.04 (1)	2.914 (4)	163 (3)
N15—H15*A*⋯O3	0.90 (1)	2.00 (2)	2.813 (4)	150 (3)

Symmetry codes: (i) 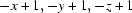; (ii) 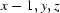; (iii) 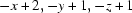; (iv) 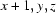; (v) 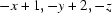; (vi) 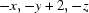.

**Fig. 1.**
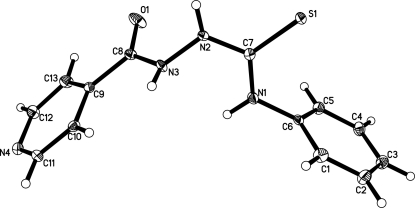
The molecular structure of the C1 molecule in (I). Displacement ellipsoids are drawn at the 30% probability level and H atoms are shown as spheres of arbitrary radius.

**Fig. 2.**
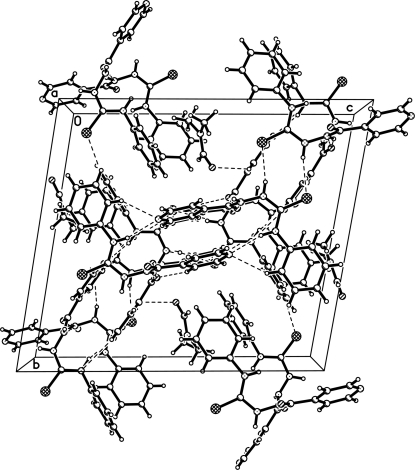
The crystal packing for (I).

## supplementary crystallographic information

### Experimental

Isonicotinohydrazide (0.1 mol) was dissolved in ethanol(30 ml), and isothiocyanatobenzene (0.1 mol) was added dropwised to it. The reaction mixture was refluxed for 4 h. After the reaction is over, cooled, the product was filtered, washed with EtOH, dried. The compound was recrystallized in DMF to obtain colourless prisms of (I).

### Refinement

All the H atoms were positioned geometrically (C—H = 0.93–0.97 Å) and refined as riding with *U*_iso_(H) = 1.2*U*_eq_(C) or 1.5*U*_eq_(methyl C).

### Crystal data

**Table:**

2C_13_H_12_N_4_OS·C_3_H_7_NO	*Z* = 4
*M**_r_* = 617.74	*F*(000) = 1296
Triclinic, *P*1	*D*_x_ = 1.371 Mg m^−^^3^
Hall symbol: -P 1	Mo *K*α radiation, λ = 0.71073 Å
*a* = 9.901 (3) Å	Cell parameters from 8688 reflections
*b* = 16.583 (5) Å	θ = 1.3–27.2°
*c* = 18.610 (5) Å	µ = 0.23 mm^−^^1^
α = 99.350 (6)°	*T* = 113 K
β = 91.831 (6)°	Prism, colorless
γ = 96.454 (4)°	0.22 × 0.20 × 0.10 mm
*V* = 2991.8 (15) Å^3^	

### Data collection

**Table:**

Rigaku Saturn CCD diffractometer	10511 independent reflections
Radiation source: rotating anode	8262 reflections with *I* > 2σ(*I*)
multilayer	*R*_int_ = 0.053
Detector resolution: 14.63 pixels mm^-1^	θ_max_ = 25.0°, θ_min_ = 1.3°
ω and φ scans	*h* = −11→11
Absorption correction: multi-scan (*CrystalClear*; Rigaku/MSC, 2005)	*k* = −19→19
*T*_min_ = 0.952, *T*_max_ = 0.978	*l* = −21→22
28071 measured reflections	

### Refinement

**Table:**

Refinement on *F*^2^	Primary atom site location: structure-invariant direct methods
Least-squares matrix: full	Secondary atom site location: difference Fourier map
*R*[*F*^2^ > 2σ(*F*^2^)] = 0.069	Hydrogen site location: inferred from neighbouring sites
*wR*(*F*^2^) = 0.143	H atoms treated by a mixture of independent and constrained refinement
*S* = 1.13	*w* = 1/[σ^2^(*F*_o_^2^) + (0.0421*P*)^2^ + 1.586*P*] where *P* = (*F*_o_^2^ + 2*F*_c_^2^)/3
10511 reflections	(Δ/σ)_max_ = 0.004
827 parameters	Δρ_max_ = 0.80 e Å^−^^3^
12 restraints	Δρ_min_ = −0.62 e Å^−^^3^

### Special details

**Table:**

Geometry. All e.s.d.'s (except the e.s.d. in the dihedral angle between two l.s. planes) are estimated using the full covariance matrix. The cell e.s.d.'s are taken into account individually in the estimation of e.s.d.'s in distances, angles and torsion angles; correlations between e.s.d.'s in cell parameters are only used when they are defined by crystal symmetry. An approximate (isotropic) treatment of cell e.s.d.'s is used for estimating e.s.d.'s involving l.s. planes.
Refinement. Refinement of *F*^2^ against ALL reflections. The weighted *R*-factor *wR* and goodness of fit *S* are based on *F*^2^, conventional *R*-factors *R* are based on *F*, with *F* set to zero for negative *F*^2^. The threshold expression of *F*^2^ > σ(*F*^2^) is used only for calculating *R*-factors(gt) *etc*. and is not relevant to the choice of reflections for refinement. *R*-factors based on *F*^2^ are statistically about twice as large as those based on *F*, and *R*- factors based on ALL data will be even larger.

### Fractional atomic coordinates and isotropic or equivalent isotropic displacement parameters (Å^2^)

**Table:**

	*x*	*y*	*z*	*U*_iso_*/*U*_eq_	
S1	0.42825 (8)	0.39646 (5)	0.41270 (5)	0.0189 (2)	
S2	0.94449 (9)	0.63964 (5)	0.15999 (5)	0.0208 (2)	
S3	0.58024 (9)	1.10379 (5)	0.08399 (5)	0.0197 (2)	
S4	0.10408 (8)	0.84954 (5)	0.33057 (5)	0.0204 (2)	
O1	0.7224 (2)	0.65592 (15)	0.40063 (15)	0.0339 (7)	
O2	1.2090 (2)	0.60713 (15)	0.37207 (12)	0.0284 (6)	
O3	0.2916 (2)	0.83621 (14)	0.09366 (13)	0.0247 (6)	
O4	−0.1961 (2)	0.88978 (14)	0.13020 (12)	0.0223 (5)	
N1	0.4231 (3)	0.48085 (16)	0.30234 (15)	0.0182 (6)	
N2	0.4964 (3)	0.55465 (16)	0.41441 (15)	0.0182 (6)	
N3	0.4948 (3)	0.62797 (16)	0.38738 (15)	0.0166 (6)	
N4	0.5991 (3)	0.89327 (16)	0.28566 (15)	0.0211 (7)	
N5	0.8970 (3)	0.52016 (16)	0.24289 (15)	0.0167 (6)	
N6	0.9946 (3)	0.64832 (16)	0.29869 (15)	0.0181 (6)	
N7	0.9869 (3)	0.62694 (17)	0.36793 (15)	0.0176 (6)	
N8	1.0703 (3)	0.56504 (16)	0.62297 (14)	0.0187 (6)	
N9	0.6011 (3)	1.02115 (16)	0.19514 (15)	0.0171 (6)	
N10	0.5095 (3)	0.94706 (16)	0.08614 (15)	0.0168 (6)	
N11	0.5179 (3)	0.87375 (16)	0.11220 (15)	0.0165 (6)	
N12	0.4464 (3)	0.60558 (16)	0.20864 (15)	0.0215 (7)	
N13	0.1000 (3)	0.97489 (16)	0.25357 (15)	0.0174 (6)	
N14	0.0278 (3)	0.84359 (16)	0.19434 (15)	0.0182 (6)	
N15	0.0258 (3)	0.86872 (16)	0.12661 (15)	0.0167 (6)	
N16	−0.0810 (3)	0.93710 (16)	−0.12310 (15)	0.0189 (6)	
C1	0.2354 (3)	0.3870 (2)	0.23617 (18)	0.0238 (8)	
H1	0.1728	0.4217	0.2582	0.029*	
C2	0.1886 (4)	0.3164 (2)	0.18720 (19)	0.0271 (9)	
H2	0.0938	0.3026	0.1756	0.032*	
C3	0.2801 (4)	0.2661 (2)	0.15526 (19)	0.0253 (8)	
H3	0.2478	0.2175	0.1219	0.030*	
C4	0.4172 (3)	0.2859 (2)	0.17148 (18)	0.0235 (8)	
H4	0.4796	0.2513	0.1492	0.028*	
C5	0.4646 (3)	0.3564 (2)	0.22046 (18)	0.0213 (8)	
H5	0.5595	0.3702	0.2317	0.026*	
C6	0.3736 (3)	0.40670 (19)	0.25297 (17)	0.0159 (7)	
C7	0.4493 (3)	0.48185 (19)	0.37278 (18)	0.0151 (7)	
C8	0.6117 (3)	0.67587 (19)	0.38311 (18)	0.0175 (7)	
C9	0.6019 (3)	0.75378 (19)	0.35282 (17)	0.0155 (7)	
C10	0.4807 (3)	0.78500 (19)	0.33940 (17)	0.0174 (7)	
H10	0.3966	0.7593	0.3526	0.021*	
C11	0.4849 (3)	0.85410 (19)	0.30657 (18)	0.0201 (8)	
H11	0.4015	0.8752	0.2983	0.024*	
C12	0.7153 (3)	0.8633 (2)	0.30006 (19)	0.0237 (8)	
H12	0.7979	0.8902	0.2863	0.028*	
C13	0.7215 (3)	0.7957 (2)	0.33373 (19)	0.0243 (8)	
H13	0.8069	0.7778	0.3438	0.029*	
C14	0.7726 (3)	0.4634 (2)	0.12343 (19)	0.0227 (8)	
H14	0.7607	0.5169	0.1143	0.027*	
C15	0.7227 (3)	0.3944 (2)	0.07284 (19)	0.0250 (8)	
H15	0.6793	0.4014	0.0284	0.030*	
C16	0.7352 (3)	0.3155 (2)	0.08605 (19)	0.0242 (8)	
H16	0.7021	0.2688	0.0507	0.029*	
C17	0.7966 (3)	0.3060 (2)	0.15150 (18)	0.0225 (8)	
H17	0.8024	0.2523	0.1620	0.027*	
C18	0.8497 (3)	0.3741 (2)	0.20196 (19)	0.0206 (8)	
H18	0.8927	0.3668	0.2464	0.025*	
C19	0.8402 (3)	0.45344 (19)	0.18772 (18)	0.0177 (7)	
C20	0.9432 (3)	0.59821 (19)	0.23699 (17)	0.0153 (7)	
C21	1.1028 (3)	0.61355 (19)	0.40321 (18)	0.0173 (7)	
C22	1.0885 (3)	0.60278 (19)	0.48151 (17)	0.0160 (7)	
C23	0.9716 (3)	0.61211 (19)	0.51962 (18)	0.0196 (8)	
H23	0.8956	0.6312	0.4982	0.023*	
C24	0.9672 (3)	0.5933 (2)	0.58929 (18)	0.0213 (8)	
H24	0.8869	0.6006	0.6149	0.026*	
C25	1.1834 (3)	0.5593 (2)	0.58668 (19)	0.0221 (8)	
H25	1.2590	0.5417	0.6100	0.027*	
C26	1.1978 (3)	0.5773 (2)	0.51724 (18)	0.0216 (8)	
H26	1.2814	0.5724	0.4941	0.026*	
C27	0.8007 (3)	1.0963 (2)	0.26484 (18)	0.0212 (8)	
H27	0.8512	1.0524	0.2473	0.025*	
C28	0.8616 (3)	1.1642 (2)	0.3137 (2)	0.0269 (9)	
H28	0.9548	1.1671	0.3288	0.032*	
C29	0.7879 (3)	1.2275 (2)	0.34027 (19)	0.0242 (8)	
H29	0.8295	1.2729	0.3746	0.029*	
C30	0.6528 (3)	1.2244 (2)	0.31673 (19)	0.0238 (8)	
H30	0.6022	1.2683	0.3344	0.029*	
C31	0.5914 (3)	1.1576 (2)	0.26745 (18)	0.0205 (8)	
H31	0.4991	1.1556	0.2511	0.025*	
C32	0.6658 (3)	1.09362 (19)	0.24214 (17)	0.0157 (7)	
C33	0.5633 (3)	1.02003 (19)	0.12556 (17)	0.0144 (7)	
C34	0.4044 (3)	0.82086 (19)	0.11223 (17)	0.0165 (7)	
C35	0.4240 (3)	0.74300 (18)	0.14083 (17)	0.0139 (7)	
C36	0.5480 (3)	0.71224 (19)	0.14675 (18)	0.0192 (8)	
H36	0.6272	0.7373	0.1279	0.023*	
C37	0.5537 (3)	0.6444 (2)	0.18060 (18)	0.0223 (8)	
H37	0.6391	0.6239	0.1843	0.027*	
C38	0.3270 (3)	0.6345 (2)	0.20068 (19)	0.0237 (8)	
H38	0.2490	0.6073	0.2189	0.028*	
C39	0.3108 (3)	0.7017 (2)	0.16744 (18)	0.0223 (8)	
H39	0.2235	0.7195	0.1628	0.027*	
C40	0.2161 (3)	1.1113 (2)	0.29301 (19)	0.0211 (8)	
H40	0.2443	1.1103	0.2446	0.025*	
C41	0.2497 (3)	1.1818 (2)	0.3448 (2)	0.0264 (8)	
H41	0.2994	1.2290	0.3315	0.032*	
C42	0.2107 (3)	1.1833 (2)	0.4158 (2)	0.0289 (9)	
H42	0.2341	1.2312	0.4514	0.035*	
C43	0.1369 (4)	1.1136 (2)	0.4343 (2)	0.0283 (9)	
H43	0.1110	1.1142	0.4831	0.034*	
C44	0.1006 (3)	1.0434 (2)	0.38276 (19)	0.0239 (8)	
H44	0.0485	0.9969	0.3960	0.029*	
C45	0.1408 (3)	1.04159 (19)	0.31175 (18)	0.0184 (7)	
C46	0.0769 (3)	0.89406 (19)	0.25652 (18)	0.0162 (7)	
C47	−0.0932 (3)	0.88310 (18)	0.09519 (18)	0.0163 (7)	
C48	−0.0867 (3)	0.89707 (19)	0.01753 (17)	0.0161 (7)	
C49	0.0158 (3)	0.87464 (19)	−0.02876 (18)	0.0189 (7)	
H49	0.0854	0.8454	−0.0132	0.023*	
C50	0.0143 (3)	0.89576 (19)	−0.09763 (18)	0.0199 (8)	
H50	0.0845	0.8802	−0.1287	0.024*	
C51	−0.1810 (3)	0.95574 (19)	−0.07945 (18)	0.0200 (8)	
H51	−0.2511	0.9831	−0.0972	0.024*	
C52	−0.1884 (3)	0.93733 (19)	−0.00912 (18)	0.0185 (7)	
H52	−0.2616	0.9520	0.0201	0.022*	
O5	0.2101 (3)	0.27365 (17)	−0.02783 (15)	0.0466 (8)	
O6	0.6906 (4)	0.2373 (2)	0.52286 (19)	0.0914 (15)	
N17	0.2070 (3)	0.40408 (18)	0.03378 (16)	0.0263 (7)	
N18	0.6211 (4)	0.1077 (2)	0.46409 (18)	0.0476 (10)	
C53	0.1487 (4)	0.3305 (3)	0.0022 (2)	0.0396 (11)	
H53	0.0524	0.3203	0.0025	0.047*	
C54	0.1290 (4)	0.4666 (2)	0.0685 (2)	0.0432 (11)	
H54A	0.0317	0.4467	0.0612	0.065*	
H54B	0.1481	0.5166	0.0469	0.065*	
H54C	0.1542	0.4790	0.1208	0.065*	
C55	0.3549 (4)	0.4229 (2)	0.0394 (2)	0.0380 (10)	
H55A	0.3965	0.3726	0.0229	0.057*	
H55B	0.3848	0.4445	0.0903	0.057*	
H55C	0.3827	0.4641	0.0089	0.057*	
C56	0.5995 (6)	0.1852 (4)	0.4859 (3)	0.0741 (17)	
H56	0.5145	0.2023	0.4736	0.089*	
C57	0.7430 (5)	0.0727 (4)	0.4714 (3)	0.088 (2)	
H57A	0.8156	0.1157	0.4929	0.133*	
H57B	0.7682	0.0469	0.4233	0.133*	
H57C	0.7300	0.0310	0.5032	0.133*	
C58	0.5119 (5)	0.0544 (3)	0.4171 (3)	0.0615 (14)	
H58A	0.4290	0.0817	0.4186	0.092*	
H58B	0.4937	0.0018	0.4347	0.092*	
H58C	0.5406	0.0443	0.3668	0.092*	
H1A	0.440 (3)	0.5255 (14)	0.2809 (17)	0.034 (11)*	
H2A	0.516 (3)	0.562 (2)	0.4626 (6)	0.023 (10)*	
H3A	0.416 (2)	0.649 (2)	0.388 (2)	0.046 (12)*	
H5A	0.914 (3)	0.5047 (19)	0.2861 (10)	0.026 (10)*	
H6A	1.027 (3)	0.7011 (9)	0.2992 (19)	0.032 (11)*	
H7A	0.912 (2)	0.636 (2)	0.3921 (18)	0.043 (12)*	
H9A	0.593 (3)	0.9749 (12)	0.2148 (17)	0.025 (10)*	
H10A	0.488 (4)	0.939 (2)	0.0379 (7)	0.048 (13)*	
H11A	0.6012 (18)	0.858 (2)	0.117 (2)	0.047 (13)*	
H13A	0.087 (3)	0.9906 (19)	0.2101 (10)	0.022 (10)*	
H14A	−0.001 (3)	0.7901 (8)	0.193 (2)	0.037 (11)*	
H15A	0.099 (2)	0.862 (2)	0.1003 (17)	0.046 (12)*	

### Atomic displacement parameters (Å^2^)

**Table:**

	*U*^11^	*U*^22^	*U*^33^	*U*^12^	*U*^13^	*U*^23^
S1	0.0252 (5)	0.0141 (4)	0.0175 (5)	0.0000 (3)	−0.0011 (4)	0.0052 (3)
S2	0.0260 (5)	0.0195 (5)	0.0166 (5)	−0.0032 (4)	−0.0014 (4)	0.0067 (4)
S3	0.0299 (5)	0.0130 (4)	0.0164 (5)	0.0017 (3)	0.0000 (4)	0.0038 (3)
S4	0.0244 (5)	0.0190 (5)	0.0185 (5)	0.0013 (3)	−0.0024 (4)	0.0069 (4)
O1	0.0147 (13)	0.0386 (16)	0.0558 (19)	0.0074 (11)	0.0015 (12)	0.0273 (14)
O2	0.0182 (13)	0.0485 (17)	0.0203 (14)	0.0080 (11)	0.0026 (11)	0.0083 (12)
O3	0.0131 (12)	0.0266 (14)	0.0380 (16)	0.0035 (10)	0.0001 (11)	0.0155 (12)
O4	0.0172 (12)	0.0300 (14)	0.0208 (14)	0.0065 (10)	0.0045 (10)	0.0045 (11)
N1	0.0249 (16)	0.0142 (16)	0.0159 (17)	0.0043 (12)	0.0019 (12)	0.0024 (12)
N2	0.0268 (16)	0.0145 (15)	0.0134 (17)	0.0006 (12)	−0.0032 (13)	0.0048 (12)
N3	0.0164 (15)	0.0135 (15)	0.0220 (17)	0.0023 (12)	0.0023 (12)	0.0085 (12)
N4	0.0239 (16)	0.0177 (15)	0.0212 (17)	0.0018 (12)	−0.0007 (13)	0.0025 (13)
N5	0.0200 (15)	0.0169 (15)	0.0128 (16)	−0.0012 (11)	−0.0024 (12)	0.0046 (12)
N6	0.0257 (16)	0.0153 (16)	0.0133 (16)	−0.0004 (12)	−0.0027 (12)	0.0050 (12)
N7	0.0167 (15)	0.0215 (16)	0.0157 (16)	0.0033 (12)	0.0017 (12)	0.0060 (12)
N8	0.0236 (16)	0.0155 (15)	0.0147 (16)	−0.0017 (12)	−0.0024 (12)	−0.0004 (12)
N9	0.0231 (16)	0.0124 (15)	0.0165 (16)	0.0014 (11)	0.0003 (12)	0.0054 (12)
N10	0.0237 (16)	0.0110 (15)	0.0160 (17)	0.0000 (11)	−0.0040 (12)	0.0050 (12)
N11	0.0177 (16)	0.0118 (14)	0.0209 (16)	0.0018 (12)	0.0001 (12)	0.0053 (12)
N12	0.0284 (17)	0.0161 (15)	0.0201 (17)	0.0023 (12)	0.0010 (13)	0.0033 (12)
N13	0.0200 (15)	0.0177 (16)	0.0145 (16)	0.0019 (11)	−0.0009 (12)	0.0034 (12)
N14	0.0232 (16)	0.0139 (16)	0.0175 (16)	−0.0008 (12)	−0.0003 (12)	0.0049 (13)
N15	0.0173 (15)	0.0200 (15)	0.0138 (16)	0.0018 (12)	0.0032 (12)	0.0060 (12)
N16	0.0216 (15)	0.0155 (15)	0.0183 (16)	−0.0006 (12)	0.0000 (12)	0.0012 (12)
C1	0.0216 (19)	0.027 (2)	0.023 (2)	0.0067 (15)	0.0038 (15)	0.0011 (16)
C2	0.022 (2)	0.027 (2)	0.030 (2)	−0.0010 (15)	−0.0016 (16)	0.0012 (17)
C3	0.034 (2)	0.022 (2)	0.018 (2)	−0.0011 (16)	−0.0008 (16)	0.0010 (15)
C4	0.028 (2)	0.0177 (19)	0.024 (2)	0.0044 (15)	0.0043 (16)	0.0004 (16)
C5	0.0186 (18)	0.0191 (19)	0.026 (2)	0.0033 (14)	0.0004 (15)	0.0024 (15)
C6	0.0236 (18)	0.0133 (17)	0.0112 (18)	0.0010 (13)	−0.0002 (14)	0.0046 (14)
C7	0.0117 (16)	0.0156 (17)	0.0183 (19)	0.0027 (13)	0.0031 (13)	0.0028 (14)
C8	0.0183 (18)	0.0190 (18)	0.0161 (19)	0.0024 (14)	0.0013 (14)	0.0060 (14)
C9	0.0196 (18)	0.0150 (17)	0.0121 (18)	0.0020 (13)	0.0011 (13)	0.0024 (14)
C10	0.0162 (17)	0.0184 (18)	0.0167 (19)	−0.0014 (13)	−0.0010 (14)	0.0034 (14)
C11	0.0224 (19)	0.0167 (18)	0.022 (2)	0.0039 (14)	−0.0018 (15)	0.0047 (15)
C12	0.0197 (19)	0.022 (2)	0.029 (2)	−0.0025 (15)	0.0028 (15)	0.0093 (16)
C13	0.0172 (18)	0.027 (2)	0.029 (2)	0.0019 (15)	−0.0004 (15)	0.0082 (17)
C14	0.0224 (19)	0.0187 (19)	0.027 (2)	0.0002 (14)	−0.0043 (15)	0.0079 (16)
C15	0.028 (2)	0.025 (2)	0.022 (2)	−0.0016 (15)	−0.0035 (16)	0.0070 (16)
C16	0.0233 (19)	0.021 (2)	0.025 (2)	−0.0040 (15)	0.0023 (15)	−0.0012 (16)
C17	0.028 (2)	0.0155 (18)	0.025 (2)	0.0042 (14)	0.0025 (16)	0.0042 (15)
C18	0.0207 (18)	0.0215 (19)	0.021 (2)	0.0037 (14)	0.0005 (14)	0.0066 (15)
C19	0.0147 (17)	0.0169 (18)	0.022 (2)	0.0004 (13)	0.0031 (14)	0.0042 (15)
C20	0.0099 (16)	0.0170 (18)	0.0195 (19)	0.0011 (13)	−0.0005 (13)	0.0048 (14)
C21	0.0172 (18)	0.0180 (18)	0.0165 (19)	0.0020 (14)	0.0006 (14)	0.0021 (14)
C22	0.0166 (17)	0.0139 (17)	0.0161 (19)	−0.0004 (13)	−0.0016 (13)	0.0003 (14)
C23	0.0182 (18)	0.0226 (19)	0.019 (2)	0.0057 (14)	−0.0025 (14)	0.0045 (15)
C24	0.0189 (18)	0.027 (2)	0.0170 (19)	0.0033 (15)	0.0041 (14)	−0.0008 (15)
C25	0.0200 (19)	0.0213 (19)	0.024 (2)	0.0010 (14)	−0.0051 (15)	0.0046 (16)
C26	0.0171 (18)	0.028 (2)	0.020 (2)	0.0057 (15)	0.0030 (14)	0.0034 (16)
C27	0.0210 (18)	0.0185 (19)	0.025 (2)	0.0068 (14)	0.0003 (15)	0.0043 (15)
C28	0.0199 (19)	0.028 (2)	0.031 (2)	−0.0008 (15)	−0.0058 (16)	0.0048 (17)
C29	0.032 (2)	0.0175 (19)	0.021 (2)	−0.0034 (15)	−0.0038 (16)	0.0033 (15)
C30	0.033 (2)	0.0175 (19)	0.021 (2)	0.0066 (15)	0.0024 (16)	0.0022 (15)
C31	0.0205 (18)	0.0205 (19)	0.021 (2)	0.0059 (14)	0.0028 (14)	0.0017 (15)
C32	0.0193 (18)	0.0161 (17)	0.0116 (18)	−0.0006 (13)	0.0038 (13)	0.0035 (14)
C33	0.0118 (16)	0.0147 (17)	0.0172 (19)	0.0043 (13)	0.0017 (13)	0.0021 (14)
C34	0.0156 (18)	0.0173 (18)	0.0156 (18)	0.0000 (14)	0.0011 (13)	0.0008 (14)
C35	0.0170 (17)	0.0119 (17)	0.0129 (18)	0.0011 (13)	−0.0003 (13)	0.0026 (13)
C36	0.0172 (18)	0.0176 (18)	0.024 (2)	0.0020 (14)	0.0069 (14)	0.0047 (15)
C37	0.0259 (19)	0.0185 (19)	0.023 (2)	0.0083 (15)	0.0026 (15)	0.0021 (15)
C38	0.0223 (19)	0.0211 (19)	0.027 (2)	−0.0061 (15)	0.0001 (15)	0.0087 (16)
C39	0.0196 (18)	0.0219 (19)	0.026 (2)	−0.0002 (14)	−0.0002 (15)	0.0092 (16)
C40	0.0223 (19)	0.0218 (19)	0.020 (2)	0.0033 (14)	−0.0007 (15)	0.0048 (15)
C41	0.0189 (19)	0.025 (2)	0.034 (2)	−0.0004 (15)	−0.0024 (16)	0.0024 (17)
C42	0.024 (2)	0.028 (2)	0.032 (2)	0.0073 (16)	−0.0077 (17)	−0.0044 (17)
C43	0.032 (2)	0.035 (2)	0.018 (2)	0.0108 (17)	−0.0006 (16)	0.0004 (17)
C44	0.029 (2)	0.022 (2)	0.022 (2)	0.0072 (15)	0.0022 (15)	0.0041 (16)
C45	0.0186 (18)	0.0179 (18)	0.0185 (19)	0.0042 (14)	−0.0025 (14)	0.0018 (15)
C46	0.0079 (16)	0.0200 (18)	0.0213 (19)	0.0024 (13)	0.0014 (13)	0.0044 (15)
C47	0.0176 (18)	0.0120 (17)	0.0193 (19)	0.0011 (13)	0.0009 (14)	0.0034 (14)
C48	0.0174 (17)	0.0129 (17)	0.0154 (18)	−0.0024 (13)	−0.0021 (13)	−0.0021 (14)
C49	0.0184 (18)	0.0163 (18)	0.023 (2)	0.0054 (14)	0.0022 (14)	0.0042 (15)
C50	0.0224 (19)	0.0176 (18)	0.020 (2)	0.0033 (14)	0.0071 (15)	0.0008 (15)
C51	0.0194 (18)	0.0188 (18)	0.023 (2)	0.0024 (14)	−0.0009 (15)	0.0061 (15)
C52	0.0131 (17)	0.0216 (18)	0.022 (2)	0.0041 (13)	0.0044 (14)	0.0055 (15)
O5	0.076 (2)	0.0296 (17)	0.0307 (17)	−0.0064 (15)	0.0082 (15)	0.0035 (14)
O6	0.143 (4)	0.075 (3)	0.042 (2)	−0.047 (3)	0.020 (2)	0.005 (2)
N17	0.0270 (17)	0.0284 (18)	0.0227 (18)	0.0015 (13)	−0.0013 (13)	0.0034 (14)
N18	0.094 (3)	0.0207 (19)	0.029 (2)	0.0070 (19)	0.020 (2)	0.0020 (16)
C53	0.047 (3)	0.047 (3)	0.024 (2)	−0.009 (2)	−0.0014 (19)	0.016 (2)
C54	0.052 (3)	0.044 (3)	0.038 (3)	0.017 (2)	0.009 (2)	0.011 (2)
C55	0.035 (2)	0.035 (2)	0.044 (3)	0.0019 (18)	0.0021 (19)	0.010 (2)
C56	0.105 (5)	0.081 (4)	0.042 (3)	0.010 (4)	0.033 (3)	0.020 (3)
C57	0.050 (3)	0.161 (6)	0.077 (4)	0.028 (4)	0.018 (3)	0.074 (4)
C58	0.071 (4)	0.067 (4)	0.050 (3)	0.006 (3)	0.006 (3)	0.022 (3)

### Geometric parameters (Å, °)

**Table:**

S1—C7	1.699 (3)	C17—C18	1.387 (4)
S2—C20	1.686 (3)	C17—H17	0.9500
S3—C33	1.691 (3)	C18—C19	1.396 (4)
S4—C46	1.694 (3)	C18—H18	0.9500
O1—C8	1.229 (4)	C21—C22	1.506 (4)
O2—C21	1.223 (4)	C22—C23	1.387 (4)
O3—C34	1.224 (4)	C22—C26	1.393 (4)
O4—C47	1.232 (4)	C23—C24	1.383 (4)
N1—C7	1.325 (4)	C23—H23	0.9500
N1—C6	1.437 (4)	C24—H24	0.9500
N1—H1A	0.898 (10)	C25—C26	1.381 (5)
N2—C7	1.349 (4)	C25—H25	0.9500
N2—N3	1.391 (4)	C26—H26	0.9500
N2—H2A	0.897 (10)	C27—C32	1.381 (4)
N3—C8	1.341 (4)	C27—C28	1.392 (4)
N3—H3A	0.895 (10)	C27—H27	0.9500
N4—C12	1.339 (4)	C28—C29	1.380 (5)
N4—C11	1.340 (4)	C28—H28	0.9500
N5—C20	1.346 (4)	C29—C30	1.387 (5)
N5—C19	1.431 (4)	C29—H29	0.9500
N5—H5A	0.899 (10)	C30—C31	1.386 (4)
N6—C20	1.351 (4)	C30—H30	0.9500
N6—N7	1.393 (4)	C31—C32	1.387 (4)
N6—H6A	0.896 (10)	C31—H31	0.9500
N7—C21	1.361 (4)	C34—C35	1.504 (4)
N7—H7A	0.895 (10)	C35—C36	1.389 (4)
N8—C25	1.331 (4)	C35—C39	1.395 (4)
N8—C24	1.347 (4)	C36—C37	1.381 (4)
N9—C33	1.333 (4)	C36—H36	0.9500
N9—C32	1.439 (4)	C37—H37	0.9500
N9—H9A	0.898 (10)	C38—C39	1.381 (4)
N10—C33	1.352 (4)	C38—H38	0.9500
N10—N11	1.390 (3)	C39—H39	0.9500
N10—H10A	0.901 (10)	C40—C41	1.390 (4)
N11—C34	1.346 (4)	C40—C45	1.402 (4)
N11—H11A	0.897 (10)	C40—H40	0.9500
N12—C38	1.337 (4)	C41—C42	1.385 (5)
N12—C37	1.345 (4)	C41—H41	0.9500
N13—C46	1.344 (4)	C42—C43	1.394 (5)
N13—C45	1.429 (4)	C42—H42	0.9500
N13—H13A	0.899 (10)	C43—C44	1.387 (5)
N14—C46	1.354 (4)	C43—H43	0.9500
N14—N15	1.391 (4)	C44—C45	1.389 (5)
N14—H14A	0.897 (10)	C44—H44	0.9500
N15—C47	1.360 (4)	C47—C48	1.503 (4)
N15—H15A	0.895 (10)	C48—C52	1.390 (4)
N16—C51	1.332 (4)	C48—C49	1.395 (4)
N16—C50	1.344 (4)	C49—C50	1.383 (4)
C1—C6	1.385 (4)	C49—H49	0.9500
C1—C2	1.385 (5)	C50—H50	0.9500
C1—H1	0.9500	C51—C52	1.394 (4)
C2—C3	1.383 (5)	C51—H51	0.9500
C2—H2	0.9500	C52—H52	0.9500
C3—C4	1.372 (5)	O5—C53	1.245 (5)
C3—H3	0.9500	O6—C56	1.275 (6)
C4—C5	1.386 (4)	N17—C53	1.324 (5)
C4—H4	0.9500	N17—C54	1.444 (5)
C5—C6	1.382 (4)	N17—C55	1.459 (4)
C5—H5	0.9500	N18—C56	1.327 (6)
C8—C9	1.503 (4)	N18—C57	1.409 (6)
C9—C13	1.390 (4)	N18—C58	1.479 (5)
C9—C10	1.390 (4)	C53—H53	0.9500
C10—C11	1.381 (4)	C54—H54A	0.9800
C10—H10	0.9500	C54—H54B	0.9800
C11—H11	0.9500	C54—H54C	0.9800
C12—C13	1.377 (4)	C55—H55A	0.9800
C12—H12	0.9500	C55—H55B	0.9800
C13—H13	0.9500	C55—H55C	0.9800
C14—C15	1.390 (4)	C56—H56	0.9500
C14—C19	1.395 (4)	C57—H57A	0.9800
C14—H14	0.9500	C57—H57B	0.9800
C15—C16	1.388 (5)	C57—H57C	0.9800
C15—H15	0.9500	C58—H58A	0.9800
C16—C17	1.384 (5)	C58—H58B	0.9800
C16—H16	0.9500	C58—H58C	0.9800
			
C7—N1—C6	122.5 (3)	C25—C26—H26	120.4
C7—N1—H1A	123 (2)	C22—C26—H26	120.4
C6—N1—H1A	114 (2)	C32—C27—C28	119.1 (3)
C7—N2—N3	120.9 (3)	C32—C27—H27	120.4
C7—N2—H2A	125 (2)	C28—C27—H27	120.4
N3—N2—H2A	114 (2)	C29—C28—C27	120.6 (3)
C8—N3—N2	119.9 (3)	C29—C28—H28	119.7
C8—N3—H3A	120 (2)	C27—C28—H28	119.7
N2—N3—H3A	116 (3)	C28—C29—C30	119.7 (3)
C12—N4—C11	116.4 (3)	C28—C29—H29	120.1
C20—N5—C19	129.6 (3)	C30—C29—H29	120.1
C20—N5—H5A	117 (2)	C31—C30—C29	120.1 (3)
C19—N5—H5A	113 (2)	C31—C30—H30	119.9
C20—N6—N7	123.8 (3)	C29—C30—H30	119.9
C20—N6—H6A	122 (2)	C30—C31—C32	119.6 (3)
N7—N6—H6A	114 (2)	C30—C31—H31	120.2
C21—N7—N6	119.0 (3)	C32—C31—H31	120.2
C21—N7—H7A	121 (2)	C27—C32—C31	120.7 (3)
N6—N7—H7A	118 (2)	C27—C32—N9	118.9 (3)
C25—N8—C24	116.2 (3)	C31—C32—N9	120.3 (3)
C33—N9—C32	123.2 (3)	N9—C33—N10	117.5 (3)
C33—N9—H9A	121 (2)	N9—C33—S3	124.0 (2)
C32—N9—H9A	115 (2)	N10—C33—S3	118.5 (2)
C33—N10—N11	121.1 (3)	O3—C34—N11	122.8 (3)
C33—N10—H10A	124 (3)	O3—C34—C35	121.8 (3)
N11—N10—H10A	113 (3)	N11—C34—C35	115.3 (3)
C34—N11—N10	119.2 (3)	C36—C35—C39	117.8 (3)
C34—N11—H11A	122 (2)	C36—C35—C34	124.8 (3)
N10—N11—H11A	117 (3)	C39—C35—C34	117.3 (3)
C38—N12—C37	116.4 (3)	C37—C36—C35	118.6 (3)
C46—N13—C45	128.4 (3)	C37—C36—H36	120.7
C46—N13—H13A	118 (2)	C35—C36—H36	120.7
C45—N13—H13A	114 (2)	N12—C37—C36	124.2 (3)
C46—N14—N15	123.1 (3)	N12—C37—H37	117.9
C46—N14—H14A	123 (2)	C36—C37—H37	117.9
N15—N14—H14A	114 (2)	N12—C38—C39	123.6 (3)
C47—N15—N14	120.4 (3)	N12—C38—H38	118.2
C47—N15—H15A	121 (2)	C39—C38—H38	118.2
N14—N15—H15A	117 (2)	C38—C39—C35	119.2 (3)
C51—N16—C50	117.0 (3)	C38—C39—H39	120.4
C6—C1—C2	119.8 (3)	C35—C39—H39	120.4
C6—C1—H1	120.1	C41—C40—C45	120.5 (3)
C2—C1—H1	120.1	C41—C40—H40	119.8
C3—C2—C1	119.9 (3)	C45—C40—H40	119.8
C3—C2—H2	120.0	C42—C41—C40	120.1 (3)
C1—C2—H2	120.0	C42—C41—H41	119.9
C4—C3—C2	120.3 (3)	C40—C41—H41	119.9
C4—C3—H3	119.8	C41—C42—C43	119.2 (3)
C2—C3—H3	119.8	C41—C42—H42	120.4
C3—C4—C5	120.0 (3)	C43—C42—H42	120.4
C3—C4—H4	120.0	C44—C43—C42	121.2 (3)
C5—C4—H4	120.0	C44—C43—H43	119.4
C6—C5—C4	120.0 (3)	C42—C43—H43	119.4
C6—C5—H5	120.0	C43—C44—C45	119.6 (3)
C4—C5—H5	120.0	C43—C44—H44	120.2
C5—C6—C1	120.0 (3)	C45—C44—H44	120.2
C5—C6—N1	119.9 (3)	C44—C45—C40	119.4 (3)
C1—C6—N1	120.0 (3)	C44—C45—N13	123.9 (3)
N1—C7—N2	117.8 (3)	C40—C45—N13	116.5 (3)
N1—C7—S1	123.4 (2)	N13—C46—N14	116.9 (3)
N2—C7—S1	118.7 (2)	N13—C46—S4	126.1 (3)
O1—C8—N3	121.9 (3)	N14—C46—S4	117.0 (2)
O1—C8—C9	121.3 (3)	O4—C47—N15	121.6 (3)
N3—C8—C9	116.8 (3)	O4—C47—C48	122.8 (3)
C13—C9—C10	117.5 (3)	N15—C47—C48	115.4 (3)
C13—C9—C8	117.9 (3)	C52—C48—C49	117.9 (3)
C10—C9—C8	124.5 (3)	C52—C48—C47	117.3 (3)
C11—C10—C9	118.7 (3)	C49—C48—C47	124.7 (3)
C11—C10—H10	120.6	C50—C49—C48	118.9 (3)
C9—C10—H10	120.6	C50—C49—H49	120.6
N4—C11—C10	124.2 (3)	C48—C49—H49	120.6
N4—C11—H11	117.9	N16—C50—C49	123.7 (3)
C10—C11—H11	117.9	N16—C50—H50	118.2
N4—C12—C13	123.6 (3)	C49—C50—H50	118.2
N4—C12—H12	118.2	N16—C51—C52	123.6 (3)
C13—C12—H12	118.2	N16—C51—H51	118.2
C12—C13—C9	119.5 (3)	C52—C51—H51	118.2
C12—C13—H13	120.2	C48—C52—C51	118.8 (3)
C9—C13—H13	120.2	C48—C52—H52	120.6
C15—C14—C19	119.5 (3)	C51—C52—H52	120.6
C15—C14—H14	120.2	C53—N17—C54	122.0 (3)
C19—C14—H14	120.2	C53—N17—C55	120.7 (3)
C16—C15—C14	121.2 (3)	C54—N17—C55	117.2 (3)
C16—C15—H15	119.4	C56—N18—C57	127.7 (5)
C14—C15—H15	119.4	C56—N18—C58	116.5 (5)
C17—C16—C15	118.9 (3)	C57—N18—C58	115.1 (4)
C17—C16—H16	120.6	O5—C53—N17	125.2 (4)
C15—C16—H16	120.6	O5—C53—H53	117.4
C16—C17—C18	120.7 (3)	N17—C53—H53	117.4
C16—C17—H17	119.7	N17—C54—H54A	109.5
C18—C17—H17	119.7	N17—C54—H54B	109.5
C17—C18—C19	120.3 (3)	H54A—C54—H54B	109.5
C17—C18—H18	119.9	N17—C54—H54C	109.5
C19—C18—H18	119.9	H54A—C54—H54C	109.5
C14—C19—C18	119.3 (3)	H54B—C54—H54C	109.5
C14—C19—N5	124.1 (3)	N17—C55—H55A	109.5
C18—C19—N5	116.6 (3)	N17—C55—H55B	109.5
N5—C20—N6	116.9 (3)	H55A—C55—H55B	109.5
N5—C20—S2	126.5 (2)	N17—C55—H55C	109.5
N6—C20—S2	116.6 (2)	H55A—C55—H55C	109.5
O2—C21—N7	121.9 (3)	H55B—C55—H55C	109.5
O2—C21—C22	122.8 (3)	O6—C56—N18	121.4 (6)
N7—C21—C22	115.3 (3)	O6—C56—H56	119.3
C23—C22—C26	117.4 (3)	N18—C56—H56	119.3
C23—C22—C21	124.7 (3)	N18—C57—H57A	109.5
C26—C22—C21	117.8 (3)	N18—C57—H57B	109.5
C24—C23—C22	119.2 (3)	H57A—C57—H57B	109.5
C24—C23—H23	120.4	N18—C57—H57C	109.5
C22—C23—H23	120.4	H57A—C57—H57C	109.5
N8—C24—C23	123.8 (3)	H57B—C57—H57C	109.5
N8—C24—H24	118.1	N18—C58—H58A	109.5
C23—C24—H24	118.1	N18—C58—H58B	109.5
N8—C25—C26	124.1 (3)	H58A—C58—H58B	109.5
N8—C25—H25	118.0	N18—C58—H58C	109.5
C26—C25—H25	118.0	H58A—C58—H58C	109.5
C25—C26—C22	119.3 (3)	H58B—C58—H58C	109.5
			
C7—N2—N3—C8	−119.5 (3)	C32—C27—C28—C29	1.2 (5)
C20—N6—N7—C21	110.2 (3)	C27—C28—C29—C30	−1.7 (5)
C33—N10—N11—C34	129.5 (3)	C28—C29—C30—C31	0.9 (5)
C46—N14—N15—C47	−105.5 (3)	C29—C30—C31—C32	0.3 (5)
C6—C1—C2—C3	−0.1 (5)	C28—C27—C32—C31	0.0 (5)
C1—C2—C3—C4	−0.4 (5)	C28—C27—C32—N9	−176.3 (3)
C2—C3—C4—C5	0.4 (5)	C30—C31—C32—C27	−0.8 (5)
C3—C4—C5—C6	0.1 (5)	C30—C31—C32—N9	175.5 (3)
C4—C5—C6—C1	−0.5 (5)	C33—N9—C32—C27	−111.0 (4)
C4—C5—C6—N1	−178.5 (3)	C33—N9—C32—C31	72.6 (4)
C2—C1—C6—C5	0.5 (5)	C32—N9—C33—N10	176.9 (3)
C2—C1—C6—N1	178.5 (3)	C32—N9—C33—S3	−2.5 (4)
C7—N1—C6—C5	−86.3 (4)	N11—N10—C33—N9	−12.5 (4)
C7—N1—C6—C1	95.8 (4)	N11—N10—C33—S3	166.9 (2)
C6—N1—C7—N2	179.5 (3)	N10—N11—C34—O3	−3.8 (5)
C6—N1—C7—S1	0.2 (4)	N10—N11—C34—C35	179.5 (3)
N3—N2—C7—N1	11.0 (4)	O3—C34—C35—C36	164.2 (3)
N3—N2—C7—S1	−169.6 (2)	N11—C34—C35—C36	−19.1 (5)
N2—N3—C8—O1	2.1 (5)	O3—C34—C35—C39	−20.0 (5)
N2—N3—C8—C9	179.6 (3)	N11—C34—C35—C39	156.7 (3)
O1—C8—C9—C13	10.5 (5)	C39—C35—C36—C37	−2.1 (5)
N3—C8—C9—C13	−167.0 (3)	C34—C35—C36—C37	173.7 (3)
O1—C8—C9—C10	−172.5 (3)	C38—N12—C37—C36	1.8 (5)
N3—C8—C9—C10	9.9 (5)	C35—C36—C37—N12	0.0 (5)
C13—C9—C10—C11	1.5 (5)	C37—N12—C38—C39	−1.5 (5)
C8—C9—C10—C11	−175.4 (3)	N12—C38—C39—C35	−0.6 (5)
C12—N4—C11—C10	−1.9 (5)	C36—C35—C39—C38	2.4 (5)
C9—C10—C11—N4	0.8 (5)	C34—C35—C39—C38	−173.7 (3)
C11—N4—C12—C13	0.6 (5)	C45—C40—C41—C42	1.0 (5)
N4—C12—C13—C9	1.7 (5)	C40—C41—C42—C43	−0.5 (5)
C10—C9—C13—C12	−2.7 (5)	C41—C42—C43—C44	−0.8 (5)
C8—C9—C13—C12	174.5 (3)	C42—C43—C44—C45	1.4 (5)
C19—C14—C15—C16	2.0 (5)	C43—C44—C45—C40	−0.9 (5)
C14—C15—C16—C17	1.1 (5)	C43—C44—C45—N13	−175.1 (3)
C15—C16—C17—C18	−2.5 (5)	C41—C40—C45—C44	−0.3 (5)
C16—C17—C18—C19	0.9 (5)	C41—C40—C45—N13	174.3 (3)
C15—C14—C19—C18	−3.6 (5)	C46—N13—C45—C44	−34.0 (5)
C15—C14—C19—N5	179.1 (3)	C46—N13—C45—C40	151.6 (3)
C17—C18—C19—C14	2.3 (5)	C45—N13—C46—N14	174.6 (3)
C17—C18—C19—N5	179.7 (3)	C45—N13—C46—S4	−5.7 (5)
C20—N5—C19—C14	−26.9 (5)	N15—N14—C46—N13	12.7 (4)
C20—N5—C19—C18	155.8 (3)	N15—N14—C46—S4	−167.0 (2)
C19—N5—C20—N6	−178.7 (3)	N14—N15—C47—O4	14.0 (5)
C19—N5—C20—S2	0.8 (5)	N14—N15—C47—C48	−170.9 (3)
N7—N6—C20—N5	−8.2 (4)	O4—C47—C48—C52	15.4 (5)
N7—N6—C20—S2	172.3 (2)	N15—C47—C48—C52	−159.7 (3)
N6—N7—C21—O2	−11.5 (5)	O4—C47—C48—C49	−166.5 (3)
N6—N7—C21—C22	171.6 (3)	N15—C47—C48—C49	18.4 (4)
O2—C21—C22—C23	177.8 (3)	C52—C48—C49—C50	2.2 (5)
N7—C21—C22—C23	−5.4 (5)	C47—C48—C49—C50	−175.9 (3)
O2—C21—C22—C26	−5.8 (5)	C51—N16—C50—C49	−2.3 (5)
N7—C21—C22—C26	171.0 (3)	C48—C49—C50—N16	0.0 (5)
C26—C22—C23—C24	−2.2 (5)	C50—N16—C51—C52	2.4 (5)
C21—C22—C23—C24	174.2 (3)	C49—C48—C52—C51	−2.1 (5)
C25—N8—C24—C23	3.1 (5)	C47—C48—C52—C51	176.2 (3)
C22—C23—C24—N8	−0.8 (5)	N16—C51—C52—C48	−0.2 (5)
C24—N8—C25—C26	−2.5 (5)	C54—N17—C53—O5	−179.1 (4)
N8—C25—C26—C22	−0.4 (5)	C55—N17—C53—O5	−3.6 (6)
C23—C22—C26—C25	2.8 (5)	C57—N18—C56—O6	−5.8 (7)
C21—C22—C26—C25	−173.9 (3)	C58—N18—C56—O6	−175.8 (4)

### Hydrogen-bond geometry (Å, °)

**Table:**

*D*—H···*A*	*D*—H	H···*A*	*D*···*A*	*D*—H···*A*
N3—H3A···O6^i^	0.90 (1)	2.63 (3)	3.325 (5)	135 (3)
N2—H2A···S1^i^	0.90 (1)	2.34 (1)	3.229 (3)	171 (3)
N3—H3A···O2^ii^	0.90 (1)	2.08 (3)	2.812 (4)	138 (3)
N14—H14A···S2^ii^	0.90 (1)	2.46 (1)	3.341 (3)	166 (3)
N5—H5A···N8^iii^	0.90 (1)	2.21 (1)	3.087 (4)	165 (3)
N6—H6A···S4^iv^	0.90 (1)	2.46 (1)	3.341 (3)	167 (3)
N11—H11A···O4^iv^	0.90 (1)	2.01 (2)	2.818 (3)	149 (3)
N10—H10A···S3^v^	0.90 (1)	2.32 (1)	3.211 (3)	170 (3)
N13—H13A···N16^vi^	0.90 (1)	2.17 (1)	3.041 (4)	163 (3)
N1—H1A···N12	0.90 (1)	2.04 (1)	2.909 (4)	164 (3)
N7—H7A···O1	0.90 (1)	1.95 (2)	2.784 (4)	155 (3)
N9—H9A···N4	0.90 (1)	2.04 (1)	2.914 (4)	163 (3)
N15—H15A···O3	0.90 (1)	2.00 (2)	2.813 (4)	150 (3)

Symmetry codes: (i) −*x*+1, −*y*+1, −*z*+1; (ii) *x*−1, *y*, *z*; (iii) −*x*+2, −*y*+1, −*z*+1; (iv) *x*+1, *y*, *z*; (v) −*x*+1, −*y*+2, −*z*; (vi) −*x*, −*y*+2, −*z*.
